# Molecular mechanisms underpinning favourable physiological adaptations to exercise prehabilitation for urological cancer surgery

**DOI:** 10.1038/s41391-023-00774-z

**Published:** 2023-12-18

**Authors:** James E. M. Blackwell, Nima Gharahdaghi, Colleen S. Deane, Matthew S. Brook, John P. Williams, Jonathan N. Lund, Philip J. Atherton, Ken Smith, Daniel J. Wilkinson, Bethan E. Phillips

**Affiliations:** 1 MRC-Versus Arthritis Centre for Musculoskeletal Ageing Research and National Institute for Health Research Nottingham Biomedical Research Centre, School of Medicine, University of Nottingham, Derby, UK; 2 Department of Surgery & Anaesthetics, Royal Derby Hospital, Derby, UK; 3 Human Development & Health, Faculty of Medicine, University of Southampton, Southampton General Hospital, Southampton, UK; 4 School of Life Sciences, University of Nottingham, Nottingham, UK

**Keywords:** Prostate cancer, Translational research

## Abstract

**Background:**

Surgery for urological cancers is associated with high complication rates and survivors commonly experience fatigue, reduced physical ability and quality of life. High-intensity interval training (HIIT) as surgical prehabilitation has been proven effective for improving the cardiorespiratory fitness (CRF) of urological cancer patients, however the mechanistic basis of this favourable adaptation is undefined. Thus, we aimed to assess the mechanisms of physiological responses to HIIT as surgical prehabilitation for urological cancer.

**Methods:**

Nineteen male patients scheduled for major urological surgery were randomised to complete 4-weeks HIIT prehabilitation (71.6 ± 0.75 years, BMI: 27.7 ± 0.9 kg·m^2^) or a no-intervention control (71.8 ± 1.1 years, BMI: 26.9 ± 1.3 kg·m^2^). Before and after the intervention period, patients underwent *m. vastus lateralis* biopsies to quantify the impact of HIIT on mitochondrial oxidative phosphorylation (OXPHOS) capacity, cumulative myofibrillar muscle protein synthesis (MPS) and anabolic, catabolic and insulin-related signalling.

**Results:**

OXPHOS capacity increased with HIIT, with increased expression of electron transport chain protein complexes (C)-II (*p* = 0.010) and III (*p* = 0.045); and a significant correlation between changes in C-I (*r* = 0.80, *p* = 0.003), C-IV (*r* = 0.75, *p* = 0.008) and C-V (*r* = 0.61, *p* = 0.046) and changes in CRF. Neither MPS (1.81 ± 0.12 to 2.04 ± 0.14%·day^−1^, *p* = 0.39) nor anabolic or catabolic proteins were upregulated by HIIT (*p* > 0.05). There was, however, an increase in phosphorylation of AS160^Thr642^ (*p* = 0.046) post-HIIT.

**Conclusions:**

A HIIT surgical prehabilitation regime, which improved the CRF of urological cancer patients, enhanced capacity for skeletal muscle OXPHOS; offering potential mechanistic explanation for this favourable adaptation. HIIT did not stimulate MPS, synonymous with the observed lack of hypertrophy. Larger trials pairing patient-centred and clinical endpoints with mechanistic investigations are required to determine the broader impacts of HIIT prehabilitation in this cohort, and to inform on future optimisation (i.e., to increase muscle mass).

## Introduction

Urological malignancy is the most common cancer in men, accounting for ~25% of all male cancers and 10% of male cancer-related deaths [1]. Surgery for urological cancers is associated with high complication rates including transfusion and/or ventilator requirements, and surgical site infections [2], and survivors commonly experience fatigue, reduced physical ability and reduced quality of life [3]. For example, even at 1 year after surgery, only 50% of radical prostatectomy survivors have returned to baseline levels of physical function [4]. In addition, post-surgical complications are associated with substantial increases in healthcare costs and reduced survivorship [5].

Although inextricably linked via the oxygen utilisation capacity of skeletal muscle, both cardiorespiratory fitness (CRF) and skeletal muscle mass (MM) are each physiological parameters known to be associated with improved post-operative outcomes [6] in surgical cancer patients. For example, across multiple cancer types including colon and liver, a pre-operative increase in anaerobic threshold ((AT); an established measure of CRF) of 1.5–2.0 ml/kg/min has been shown to be associated with a ~40% reduction in the odds of post-surgical complications [7, 8]. Additional work in colon cancer patients has also shown that both MM and quality are each predictive of numerous surgical outcomes including length of stay and 30-day mortality [9]. However, despite the significant role of both CRF and MM in the physiological resilience of surgical cancer patients, the majority of urology research exploring surgical prehabilitation has remained focussed on reducing urology-specific complications (e.g., urinary incontinence [10]).

Prehabilitation is defined as interventions to enhance the ‘physiological reserve’ patients before the stress of surgery [11]. While CRF remains the predominant endpoint in cancer prehabilitation research, interest in body composition (e.g., MM), clinical- and patient-centred outcomes is growing. Importantly, in the UK the time-window for cancer prehabilitation is curtailed by guidelines from the National Cancer Action Team who specify that first treatment (including surgery) should start within 31-days of decision to treat [12].

Based on this short time-window in which to implement prehabilitation for cancer, high-intensity interval training (HIIT; brief periods of high-intensity exertions interspersed with rest or active recovery) has come to the fore in this setting. Proven effective for improving both body composition [13] and the CRF of both healthy and clinical populations over shorter time-periods than commonly needed for traditional aerobic exercise training [14], we have recently shown that 4-weeks, 5×1-min (interspersed with 90 s recovery) HIIT on a cycle ergometer can improve the CRF of urological cancer patients prior to surgery [15]. HIIT has also shown potential to elicit muscle hypertrophy [16], an adaptation more commonly associated with resistance exercise training (RET), although evidence for this adaptation is inconsistent, especially across surgical cohorts. For example, our work in urological cancer patients showed no increase in whole-body MM with 4-weeks HIIT training prior to surgery [15].

With the majority of cancers, including urological, being associated with advancing age, many patients face the physiological challenges of cancer, including anabolic blunting [17], on a background of pre-existent sarcopenia furthering the importance of MM/functional maintenance in the peri-operative period. In non-clinical cohorts, the mechanisms by which HIIT enhances MM have been subject to intense study. For example, in a pre-clinical rodent model, HIIT-induced activation of the phosphoinositol-3 kinase-Akt–mTOR signal transduction pathway led to higher ribosomal biogenesis and muscle protein synthesis (MPS), and induced muscle hypertrophy to a greater extent than moderate-intensity training [18]. Further, in healthy older men, just a single session of HIIT was shown to increase MPS, which remained elevated for up to 48-hours [19]. Although less well studied in relation to skeletal muscle, cellular processes involved in the regulation of mitochondrial biogenesis and the electron transport chain (ETC) have been found to be dysregulated both by cancer and associated surgery [20], yet potentially ameliorated by mitochondria-based therapies [21]. This work highlights the adaptive capability of cellular systems associated with CRF [22], and therefore a potential mechanistic avenue for favourable exercise-induced adaptation.

Despite the therapeutic promise of exercise prehabilitation, the metabolic and molecular mechanisms by which HIIT is able to induce favourable physiological adaptations on a physiological background of cancer, and in particular urological cancer, are not well-established. Therefore, this study aimed to evaluate the impact of 4 weeks HIIT; which was able to elicit increases in CRF [15], on mechanistic adaptation (e.g., mitochondrial oxidative phosphorylation (OXPHOS) capacity, MPS, anabolic, catabolic and insulin signalling) in urological cancer patients awaiting surgery.

## Materials and methods

### Patient recruitment

As detailed in our prior publication [15], this study was approved by an NHS research ethics committee (REC reference: 16/EM/0075, IRAS Project ID 19141) and registered with Clinicaltrials.gov (NCT02671617). Patients identified as suitable for surgery with curative intent and with an allocated operation date that allowed potential for baseline assessment, 10 or more HIIT sessions and reassessment within 72 h before operation were invited to participate. Before inclusion, patients provided written informed consent to participate and underwent a medical screening against pre-defined inclusion and exclusion criteria including safety criteria as defined in ATS/ACCP cardiopulmonary exercise test (CPET) guidelines [23]. After baseline assessments, patients were randomised (block randomisation by age) to HIIT or a no-intervention control group.

### Study conduct

**Fig. 1:**
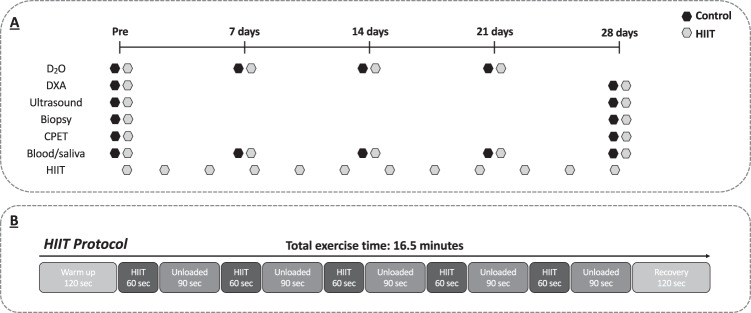
Study conduct. **A** Study design, **B** HIIT protocol. CPET cardiopulmonary exercise testing, D_2_O deuterium oxide, DXA dual energy X-ray absorptiometry, HIIT high intensity interval training.

At baseline and after the intervention period, patients had whole-body composition (body fat percentage and lean mass) measured by dual-energy X-ray absorptiometry (DXA; Lunar Prodigy II, GE Medical Systems, Little Chalfont, UK), *m. vastus lateralis* (VL) muscle architecture (muscle thickness, pennation angle and fascicle length) assessed by B-mode ultrasonography (Mylab, Esaote Biomedica, Italy), and CRF assessed by CPET (Lode Corival, Lode, Groningen) as described previously [15]. In brief, CPET was performed with in-line breath-by-breath data collected via a metabolic cart (nSpire Zan600, Germany) and anaerobic threshold (AT) interpretation conducted by two experienced assessors blinded to patient group allocation and time-point (i.e., pre- or post-intervention). A muscle biopsy of the VL was taken to quantify mitochondrial OXPHOS capacity, cumulative MPS over the intervention period and markers of anabolic, catabolic and insulin signalling. To facilitate the assessment of cumulative MPS before and after the intervention period, a blood sample was collected at the screening visit and each assessment visit. Patients then consumed 3 ml/kg deuterium oxide (D_2_O) 72-h before their first biopsy with weekly D_2_O “top-ups” (1 ml/kg) and time-matched saliva samples thereafter [24] (Fig. 1A).

The HIIT group completed up to 12 HIIT sessions (3–4 times weekly) within a < 31-day period. Each HIIT session lasted 16.5 min in total including a warm-up period of 2-min, five 1-min high-intensity exertions interspersed by 90-s unloaded cycling, and a final 2-min recovery phase (Fig. 1B).

### Laboratory analysis

#### Immunoblotting for anabolic, catabolic, insulin and mitochondrial oxidative phosphorylation markers

To prepare samples for immunoblotting, spectrophotometry was used to determine protein concentration as per our standard technique [25]. Samples (10ug) were loaded onto Criterion XT Bis–Tris–12% SDS-polyacrylamide gel electrophoresis (SDS-PAGE) gels (Bio-Rad) for electrophoresis at 185 V for 45-min, transferred onto polyvinylidene difluoride membranes blocked in 2.5% low-fat milk in TBST for 1-h at ambient temperature, and then incubated in the following primary antibodies overnight at 4 °C (1:2000 in 2.5% bovine serum albumin (BSA) in TBST): rabbit phospho-protein kinase B (Akt)^Ser473^ (#9271), phospho-AMP-activated protein kinase α (AMPKα)^Thr172^ (#2531), phospho-Akt substrate of 160 kDa (AS160)^Thr642^ (#8881), Atrogin (#AP2041), Beclin-1 (#3495), phospho-Forkhead box O3 (FOXO3a)^Ser253^ (#13129), phospho-glycogen synthase (GS)^Ser641^ (#3891), LC3B (#2775), phospho-mechanistic target of rapamycin (mTOR)^Ser2448^ (#2971), phospho-Tuberous Sclerosis Complex 2 (TSC2)^Thr1462^ (#3617), phospho-4E-BP1 (Eukaryotic translation initiation factor 4E-binding protein 1)^Thr37/46^ (#2855) (from Cell Signalling Technology, Leiden, The Netherlands), MuRF-1 (#101AP) (from ECM Biosciences, Versailles, KY, USA) and mouse OXPHOS (Abcam, Cambridge, MA, USA). After overnight incubation membranes were washed, soaked in horseradish peroxidase-conjugated secondary antibody (New England Biolabs; 1:2000 in 2.5% BSA in TBST) for 1-h and band intensity quantified (Chemidoc MP, Bio-Rad, Hemel Hempstead, UK) following exposure to Chemiluminescent HRP substrate (Millipore Corp., Billerica, MA, USA). Relative arbitrary units (AU) were normalised to coomassie-stained membranes [25].

#### Mitochondrial citrate synthase activity

Citrate synthase (CS) activity was measured as previously described [26]. Briefly, after homogenisation of 3–5 mg muscle in 1% Triton-X-100 buffer, samples were centrifuged at 22,000 *g* for 3-min and the supernatant used. Thereafter, 300 μL master mix containing 28% 0.05 M TRIS buffer (pH 7.6), 1.3% 1 mM 5,5′ dithiobis-2-nitrobenzoic acid (DTNB), 7% acetyl-coenzyme A (1.36 mg·mL^−1^), 0.8% oxaloacetate (9.88 mg·mL^−1^), and 63% ddH_2_O was measured at 412 nm as the blank. Finally, 20 μL of supernatant was used to measure the maximum rate of reaction (V max), compared with whole protein content.

#### Myofibrillar muscle protein synthesis

The saliva samples collected were processed to determine each patient’s body water enrichment over the time between biopsies to provide a measure of the precursor labelling [24] using a high-temperature conversion elemental analyser (Thermo Finnigan, Thermo Scientific, Hemel Hempstead, UK) connected to an isotope ratio mass spectrometer (Delta V advantage, Thermo Scientific). To assess protein bound alanine muscle fraction enrichment, ∼40 mg of muscle was homogenised in an ice-cold homogenisation buffer to isolate myofibrillar proteins. These were hydrolysed overnight in 0.1 M HCl and Dowex H^+^ resin at 110 °C, before elution and derivatization of the amino acids as their *n*-methoxycarbonyl methyl esters. Incorporation of deuterium into the protein bound alanine was determined by gas chromatography-pyrolysis-isotope ratio mass spectrometry (Delta V Advantage, Thermo, Hemel Hempstead, UK).

#### Calculation of fractional synthetic rate

Myofibrillar fractional synthetic rate (FSR) was calculated as follows:

$${{{{{\rm{FSR}}}}}}( \% \cdot {{{{{{\rm{day}}}}}}}^{-1})=-{{{{{\rm{In}}}}}}\left(\frac{1-\left[\frac{{\left(\right.{{{{{\rm{APE}}}}}}}_{{{{{{\rm{Ala}}}}}}}}{{{{{{{\rm{APE}}}}}}}_{{{{{{\rm{P}}}}}}}}\right]}{{{{{{\rm{t}}}}}}}\right),$$

where APE_Ala_ is deuterium enrichment of protein-bound alanine, APE_P_ is mean precursor enrichment of the body water over the period (corrected for the mean number of deuterium moieties incorporated per alanine (3.7) and dilution from the total number of hydrogens in the derivative)), and *t* is the time between the biological samples in hours (i.e., the baseline blood and first biopsy (~3 days) or between the two biopsies (<31-days)).

### Statistical analysis

Sample size was originally calculated to determine change in anaerobic threshold as detailed in our prior publication [15], however, have previously shown the ability to detect between group differences for the end points used herein with the current sample size [27]. All laboratory analysis was conducted in a single bind manner. All data passed normality testing via Kolmogorov-Smirnov. Data are expressed as mean ± SEM unless otherwise stated. Two-way analysis of variance (ANOVA) was used to compare differences at baseline and post-intervention and changes across the intervention. Correlations were assessed using Pearson’s product moment correlation coefficient. Significance was accepted as *p* < 0.05 and all statistical analyses were performed using GraphPad Prism 9.5.0 (La Jolla, CA, USA).

## Results

### Patient characteristics

Nineteen patients (HIIT: 12, control: 7) were recruited to this study, with muscle biopsies an optional aspect of our previously published study [15], hence the lower patient numbers reported herein. Baseline characteristics of the patients for this study are shown in Table 1. Patients in the HIIT group completed an average of 11 HIIT sessions with adherence (pre-determined as participation in 10 sessions or more) 92%. No adverse events were reported throughout the study.

**Table 1:** Patient baseline characteristics before a < 31-day control period (CON) or period of high-intensity interval training (HIIT).

	Control (*n* = 7)	HIIT (*n* = 12)
Age (y)	71.8 ± 1.1	71.6 ± 0.75
Weight (kg)	77.3 ± 5	80.7 ± 2.4
Body mass index (kg/m^2^)	26.9 ± 1.3	27.7 ± 0.9
Location of malignancy	Prostate	Prostate
DASI	49.97 ± 9.2	53.18 ± 9.5
CPET Wattage (W)	148 ± 37	141 ± 34
VO2PEAK (ml/kg/min)	31.98 ± 3.7	27.03 ± 3.2
VO2AT (ml/kg/min)	16.96 ± 1.9	14.15 ± 1.6
SBP (mmHg)	142 ± 9	139 ± 9
DBP (mmHg)	81 ± 6	82 ± 8

Data is mean ± SD.

*BMI* body mass index, *DASI* Dukes Activity Status Index, *CPET* cardiopulmonary exercise test, *AT* anaerobic threshold, *SBP* systolic blood pressure, *DBP* diastolic blood pressure.

### Mitochondrial oxidative phosphorylation capacity

**Fig. 2:**
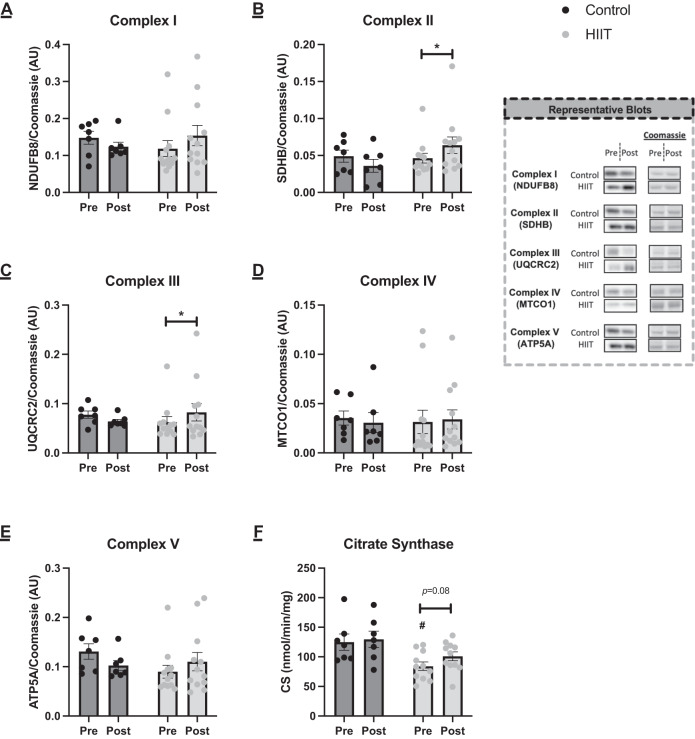
Mitochondrial oxidative phosphorylation capacity markers pre and post intervention in the control and HIIT group. **A** NDUFB8 (complex I) (group × time interaction, *p* > 0.05), **B** SDHB (complex II) (group × time interaction, *p* = 0.003), **C** UQCRC2 (complex III) (group × time interaction, *p* = 0.012), **D** MTCO1 (complex IV) (group × time interaction, *p* > 0.05), **E** ATP5A (complex V) (group × time interaction, *p* > 0.05) and **F** citrate synthase activity (group × time interaction, *p* > 0.05). Analysis via two-way ANOVA. Data is mean ± SEM. Control *n* = 7, HIIT *n* = 12 (*n* = 11 for **F**). *indicates sig*n*ificant differe*n*ce (*p* < 0.05) from pre within group; ^#^indicates significant difference (*p* < 0.05) from control at corresponding timepoint.

In keeping with our previous finding that HIIT elicited significant increases in CRF [15], HIIT resulted in enhanced protein expression of ETC complex (C)-II (*p* = 0.010) and C-III (*p* = 0.045). Protein expression for each of these complexes was not different between the groups before or after the intervention period. HIIT did not significantly enhance protein expression of C-I (*p* = 0.17), C-IV (*p* = 0.87) or C-V (*p* = 0.20) and there was no difference in protein expression between the groups at either time-point. In the control group there were no changes in ETC protein expression (C-I: *p* = 0.60, C-II: *p* = 0.17, C-III: *p* = 0.36, C-IV: *p* = 0.75, C-V: *p* = 0.17). CS activity was not altered in the HIIT (pre: 84.12 ± 7.22 vs. post: 100.93 ± 7.47 nmol/min/mg, *p* = 0.08) or control (pre: 124.83 ± 13.72 vs. post: 129.60 ± 13.80 nmol/min/mg, *p* = 0.86) groups, despite significantly lower activity in the HIIT group before the intervention period (*p* = 0.02) (Fig. 2).

**Fig. 3:**
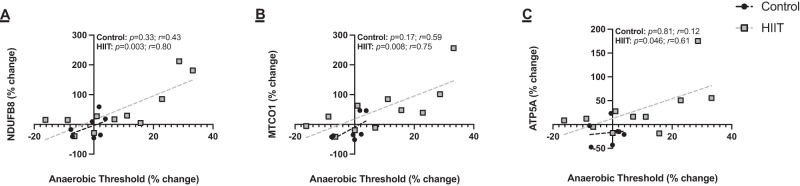
Correlations between HIIT-induced increases in cardiorespiratory fitness and changes in oxidative phosphorylation capacity. **A** NDUFB8 (complex I) and anaerobic threshold, **B** MTCO1 (complex IV) and anaerobic threshold, and **C** ATP5A (complex V) and anaerobic threshold. Analysis via Pearson’s product moment correlation coefficient. Control *n* = 7, HIIT *n* = 11.

Exploring the relationship between changes in ETC protein expression and changes in CRF, there was a significant correlation between HIIT-induced increases in CRF and increases in C-I (*r* = 0.80, *p* = 0.003), C-IV (*r* = 0.75, *p* = 0.008) and C-V (*r* = 0.61, *p* = 0.046) expression (Fig. 3). There was no relationship for C-II (*r* = 0.49, *p* = 0.13) or C-III (*r* = 0.52, *p* = 0.10). No relationships were observed in the control group (all *p* > 0.05).

### Myofibrillar muscle protein synthesis and cell signalling

**Fig. 4:**
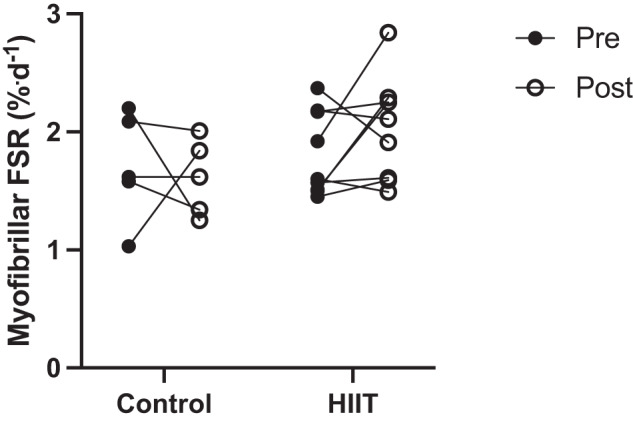
Myofibrillar fractional synthesis rate pre and post intervention in the control and HIIT group. Analysis via two-way ANOVA. Data is mean ± SEM. Control *n* = 5, HIIT *n* = 9. Group x time interaction, *p* > 0.05. FSR fractional synthetic rate.

Supporting our previously reported observation of no HIIT-induced increases in MM [15], cumulative FSR was not significantly increased by HIIT (pre: 1.81 ± 0.12 vs. post: 2.04 ± 0.14%·day^−1^, *p* = 0.39) and was not different between the HIIT and control groups either before (control: 1.70 ± 0.21 vs. HIIT: 1.81 ± 0.12%·day^−1^, *p* = 0.87) or after (control: 1.61 ± 0.14 vs. HIIT: 2.04 ± 0.14%·day^−1^, *p* = 0.13) the intervention period (Fig. 4).

Compared to baseline, HIIT significantly increased the phosphorylation of AS160^Thr642^ (*p* = 0.046) (control: AS160^Thr642^, *p* = 0.64) only (Supplementary Table S1). No other anabolic or catabolic protein markers (GS^Ser641^, mTORSer248, Akt^Ser473^; 4E-BP1^Thr37/46^, AMPKα^Thr172^, TSC2^Thr1462^, FOXO3a ^Ser253^, LC3B, Beclin-1, Atrogin, MuRF-1; all *p* > 0.05) changed in the HIIT or control groups following the intervention (Supplementary Table S1).

## Discussion

We previously reported that 4-weeks, time-efficient HIIT on a cycle ergometer as surgical prehabilitation in urological cancer positively modulated CRF [15]. This HIIT protocol involves 3 sessions per week with 5, 1-minute high-intensity exertions in each 16.5-minute session. Herein we report data that suggests this favourable physiological adaptation likely occurs due to improved mitochondrial OXPHOS capacity; thereby offering mechanistic insight into a potential strategy to offset a well-established cancer-related physiological deterioration [28], even within the short time-window available to intervene prior to surgery.

Exercise, including HIIT, is well-known to induce favourable mitochondrial adaptations. For instance, we have previously shown that in octogenarians with comorbidities, HIIT increased both muscle mitochondrial content and function [29]. Specifically related to cancer, HIIT has been shown to be a potent stimulus for counteracting reductions in muscle mitochondrial content seen in patients being treated with chemotherapy for breast cancer [30]. However, there is particularly limited data regarding muscle mitochondrial remodelling in cancer patients during the constrained time-period for prehabilitation. Here, we show that just 4-weeks HIIT improved muscle OXPHOS capacity in urological cancer patients awaiting surgery.

OXPHOS capacity has been suggested to have an important role to play in preventing the accumulation of free radicals and damaged organelles that could negatively impact muscle function [31], especially during periods of rehabilitation or recovery (i.e., from surgery). For example, Hsaio and colleagues reported a significant association between downregulation of OXPHOS capacity and an increase in fatigue in patients with prostate cancer [32]. Illustrating the spread and impact of cancer-related fatigue, it is reported to be a chronic problem in over two-thirds of patients with cancer and close to 40% of patients describe it as severe for at least 6 months after treatment [33]. Although fatigue was not assessed in this study, our results paired with the existent literature highlight the potential for HIIT prehabilitation to improve both short (i.e., physical function) and long-term (i.e., fatigue) post-surgical outcomes for urological cancer patients via preservation of mitochondrial function.

Mitochondrial biogenesis has previously been reported to be blunted by both ageing [34] and physical inactivity [35], each of which are associated with urological cancer [36]. Mitochondria have also been shown to be subject to cancer-related remodelling, including in urological cancer; with cancer patients exhibiting lower mitochondrial oxidative capacity, reduced ATP production and alterations in phospholipid metabolite profiles; all of which are indicative of mitochondrial abnormalities [37]. Thus, mitochondrial dysfunction likely underpins, at least to some extent, the poor overall physical functioning of cancer patients and presents as a potential target for interventions to improve physical function.

Although prior work has demonstrated the ability of HIIT to increase MM in healthy young and older adults [38–40], albeit with diminishing adaptation with advancing age (as is seen for RET [41]), the hypertrophic response to HIIT in cancer patients is less consistent (e.g. refs. [42, 43]). Specific to urological cancer, a systematic review by Chen et al., concluded that although exercise training can increase muscle strength it may not be sufficient to enhance MM in prostate cancer patients [44], a conclusion supported by the findings of this study. It should be noted that the review by Chen et al., was on patients undergoing androgen-deprivation therapy who were training with low systemic testosterone levels which may have blunted their hypertrophic potential [27]. That HIIT was not able to increase cumulative MPS or enhance anabolic signalling logically extrapolates to a lack of hypertrophy in this study. This does however not mirror what was seen in co-morbid octogenarians who completed the exact-same HIIT protocol [29], where increases in MPS and MM were reported, suggesting urological cancer-induced anabolic, and subsequent adaptive blunting. One possible suggestion to overcome this is the addition of RET, given its ability to elicit hypertrophy across the life course [45], including in cancer patients [46], and that hypertrophic adaptations have been shown to predominate in the early stages (3-weeks) of RET [47].

Despite a lack of change in anabolic signalling with HIIT in this study, increased phosphorylation of AS160^Thr642^ was seen. Cancer has previously been shown to lead to marked insulin resistance due to blocked insulin-stimulated glucose transport and abolished insulin-induced phosphorylation of AS160^Thr642^ at multiple phosphorylation sites [48]. As such, beyond increasing muscle mitochondrial capacity, our HIIT regime may also have a positive role to play in the glucose metabolism of urological cancer patients.

As with almost all research studies, there are limitations: 1) the majority of patients were white males with prostatic adenocarcinoma, thus representing a relatively homogenous patient group; 2) although patients were randomised to intervention group and instructed to maintain habitual physical activity and dietary intake, this was not measured in either group; 3) as only a sub-group of the original study agreed to muscle biopsies for the molecular analysis presented herein, the number of patients is relatively small which may have impacted the outcomes of our statistical analysis; and 4) our mitochondrially-focused measures only provide a measure of OXPHOS complex content, and do not represent a dynamic assessment of mitochondrial respiration, which would have required fresh-tissue analysis using (e.g.,) Oroboros or Seahorse instruments. In addition, although this study explores a pre-operative exercise intervention which could be delivered to patients with a diverse range of urological cancers, that all of the patients in this study underwent radical prostatectomy must be considered when designing future trials, as the potential post-operative impact after more invasive surgeries such as cystectomy or nephrectomy will likely differ greatly.

In conclusion, pre-operative, short-term HIIT is a well-tolerated effective intervention to improve a key aspect of physiological resilience in patients with urological cancer. This regime appears to increase CRF via improved mitochondrial oxidative capacity, whilst also enhancing glucose transport machinery. We propose this HIIT regime should now be subject to larger trials with direct clinical outcome endpoints, and potentially with the addition of adjuvant RET.

## Supplementary information

Table S1. Muscle anabolic, catabolic and insulin-related signalling responses in control and HIIT groups pre and post intervention.

## Data Availability

The datasets generated during and/or analysed during the current study are available from the corresponding author on reasonable request.
